# Evaluating the contribution of osmotic and oxidative stress components on barley growth under salt stress

**DOI:** 10.1093/aobpla/plab034

**Published:** 2021-06-11

**Authors:** Rim Nefissi Ouertani, Ghassen Abid, Chahine Karmous, Mariem Ben Chikha, Oumaima Boudaya, Henda Mahmoudi, Samiha Mejri, Robert K Jansen, Abdelwahed Ghorbel

**Affiliations:** 1 Laboratory of Plant Molecular Physiology, Center of Biotechnology of Borj Cedria, BP 901, Hammam-Lif 2050, Tunisia; 2 Laboratory of Legumes and Sustainable Agrosystems, Centre of Biotechnology of Borj Cedria, BP 901, Hammam-Lif 2050, Tunisia; 3 Laboratory of Genetics and Cereal Breeding, National Institute of Agronomy of Tunisia, Carthage University, LR14 AGR01, 1082 Tunis, Tunisia; 4 International Center for Biosaline Agriculture, P.O. Box 14660, Al Ruwayyah 2, Academic City, Dubai, United Arab Emirates; 5 Department of Integrative Biology, University of Texas at Austin, Austin, TX, USA; 6 Biotechnology Research Group, Department of Biological Sciences, Faculty of Science, King Abdulaziz, University (KAU), Jeddah 21589, Saudi Arabia

**Keywords:** Growth performance, *Hordeum vulgare*, osmoprotectants, antioxidant enzymes, *SOD* gene expression

## Abstract

Salt stress is considered one of the most devastating environmental stresses, affecting barley growth and leading to significant yield loss. Hence, there is considerable interest in investigating the most effective traits that determine barley growth under salt stress. The objective of this study was to elucidate the contribution of osmotic and oxidative stress components in leaves and roots growth under salt stress. Two distinct barley (*Hordeum vulgare*) salt-stress tolerant genotypes, Barrage Malleg (BM, tolerant) and Saouef (Sf, sensitive), were subjected to 200 mM NaCl at early vegetative stages. Stressed and control leaves and roots tissue were assessed for several growth traits, including fresh and dry weight and plant length, as well as the content of osmoprotectants proline and soluble sugars. In addition, malondialdehyde content and activities of superoxide dismutase (SOD), catalase (CAT) and ascorbate peroxidase (APX), as well as their corresponding gene expression patterns, were investigated. The results showed better performance of BM over Sf for leaf dry weight (LDW), root dry weight (RDW) and root length (RL). The salt-tolerant genotype (BM) had better osmoprotection against salt stress compared with the salt-sensitive genotype (Sf), with a higher accumulation of proline and soluble sugars in leaves and roots and a stronger antioxidant system as evidenced by higher activities of SOD, CAT and APX and more abundant *Cu/Zn-SOD* transcripts, especially in roots. Stepwise regression analysis indicated that under salt stress the most predominant trait of barley growth was *Cu/Zn-SOD* gene expression level, suggesting that alleviating oxidative stress and providing cell homeostasis is the first priority.

## Teaser

Two contrasting salt-stress tolerance barley genotypes, BM (tolerant) and Sf (sensitive), were subjected to severe salt stress. Stressed and control seedlings were assessed for several growth traits, including proline and soluble sugar content, antioxidant enzyme (SOS, CAT and APX) activities and gene expression levels. The results showed better performance of BM over Sf for growth, higher accumulation of proline and soluble sugars and stronger antioxidant system. Stepwise regression analysis indicated that under salt stress the most predominant trait of barley growth was *Cu/Zn-SOD* gene expression level, suggesting that alleviating oxidative stress and providing cell homeostasis is the first priority.

## Introduction

Salt is considered one of the most devastating environmental stresses that affects almost all crops by reducing growth components from the time of germination to maturity (Negrao *et al*. 2017), resulting in significant agricultural yield losses worldwide (Hernández 2019). This threat is increasing in arid and semi-arid areas due to the alarming rise of global temperatures. Thus, enhancing cereals’ tolerance to salt is crucial to sustain productivity.

Barley (*Hordeum vulgare*) is the fourth most important cereal crop grown for food, feed and brewing (Zhou 2010). It can be cultivated in a wide range of salt environments, making it a model system for salt-stress studies (Witzel *et al.* 2014; Schulte *et al.* 2009). In fact, barley genotypes display an extensive variability to salt-stress tolerance (Dai *et al.* 2012). Therefore, an improved knowledge of barley salt-stress tolerance mechanisms and screening for salt-tolerant barley genotypes are important for future development of this valuable crop.

Salt stress disturbs overall barley growth of leaves and roots by affecting several physiological, biochemical, and biological processes caused by molecular changes. Salt stress causes decreased turgor due to limits in leaf gas exchange and stomata closure, osmotic stress starting from roots and increased oxidative damage, all of which cause reductions in yield (Adem *et al.* 2014).

Plant responses and adaptation mechanisms to salt stress are often separated into different categories: avoidance, sensitivity and tolerance. Avoidance is a mechanism of response to salt stress (Zhao *et al.* 2020) that allows plants to sustain fundamental physiological processes by speedy stomatal closure and a reduction in total leaf area to minimize water loss by transpiration (Acosta-Motos *et al.* 2017). Decreases in leaves may provoke a reduction in height and size of aerial parts. Avoidance is also characterized by better root growth in order to increase water uptake ability. Hence, vigour allows a plant to sustain high water potential and avoid the deleterious effects of salt stress (Reddy *et al.* 2017; Allel *et al.* 2019). Root system morphology could be indicator of salt sensitivity of some plants (Acosta-Motos *et al.* 2017). For instance, less vigorous root systems with a reduced root length and branching will decrease the water and nutrient absorption capacity and induce an increased salt-stress sensitivity (Franco *et al.* 2011). In a saline environment, salt-stress sensitive genotypes exhibit irregularities during cell division, and alterations of several metabolic processes, whereas tolerant genotypes are less affected (Shahid *et al.* 2020). Indeed, tolerant plants have implemented a set of adaptations to overcome the negative effects of salt stress.

Under salt-stress conditions, tolerant genotypes tend to maintain an optimum water status by accumulating more osmoprotectants like proline and soluble sugars (Bornare *et al.* 2013). These osmoprotectants are the main actors of cellular osmotic adjustment used in maintaining cytoplasmic water content (Agarwal *et al.* 2013). Proline and soluble sugars act as salt-stress signalling compounds by regulating expression of specific genes that aid in retaining membrane integrity by preventing lipid oxidation and scavenging free radicals (Shinde *et al.* 2016).

As with other environmental stresses, salt stress induces production of plant reactive oxygen species (ROS) in both leaves and roots, such as hydroxyl-radical (•OH), superoxide-radical (O_2_^−^), hydrogen peroxide (H_2_O_2_) and singlet-oxygen (^1^O_2_), and regulate ROS homeostasis (Gupta and Huang 2014; Farnese *et al.* 2016). Above a certain threshold, ROS cause irreversible cell damages and trigger programmed cell death (Das and Roychoudhury 2014).

The increased concentration of ROS is partly balanced by antioxidant enzymatic scavenging compounds, such as superoxide dismutase (SOD), ascorbate peroxidase (APX) and catalase (CAT) (Bose *et al.* 2014). These antioxidant enzymes are the most important defence systems against oxidative stress-induced cell damage (Gupta and Huang 2014). SOD is one of the primary scavengers leading to the catalysis of the disproportionation of O_2_^•−^ radical to oxygen (O_2_) and H_2_O_2_ (Das and Roychoudhury 2014), while APX and CAT play a critical role in detoxifying plants from H_2_O_2_ accumulation that is very harmful to cell components integrity. Both antioxidant enzymes catalyse the conversion of H_2_O_2_ to water H_2_O and O_2_ (You and Chan 2015). An increase in free radicals also leads to lipid peroxidation in the cell and the overproduction of malondialdehyde (MDA), one of the final products of membrane peroxidation. MDA level is considered a reliable marker of membrane damage (Gharibi *et al.* 2016). The existence of different isoforms of antioxidant enzymes could be used as biochemical markers for stress tolerance in plants (Gill and Tuteja 2010; Zhang *et al.* 2013). In the barley genome, three SOD isozymes have been identified, copper/zinc SOD (Cu/Zn-SOD), manganese SOD (Mn-SOD) and iron SOD (Fe-SOD) (Shukla and Varma 2019). Three distinct APX isoforms have also been found in barley (Laugesen *et al.* 2007; Behrouzi *et al.* 2015). In contrast, the number of CAT isozymes in barley varied with the applied abiotic stress. Jeong and Kim (2004) reported two CAT isozymes under aluminium stress, Rohman *et al.* (2020) identified four isozymes under drought, however, only one isozyme was reported under salt stress (Behrouzi *et al.* 2015; Mohammad *et al.* 2015).

The present study was designed to investigate the interaction between transcript abundance of antioxidant components and the physiological and enzymatic components in the leaves and roots of two barley genotypes with contrasting salt-stress tolerance at the vegetative growth stage. Plants were exposed to salt stress and the analysis of growth parameters (leaf and root weight and length), proline, soluble sugars and MDA content and enzymatic activities of SOD, CAT and APX in both leaves and roots tissue were examined. Furthermore, expression patterns of antioxidant isoforms *HvCu/Zn-SOD*, *HvCAT* and *HvAPX1* were evaluated in both tissues. We also aimed to uncover the most effective traits in determination of barley growth under salt stress using stepwise regression analysis.

## Materials and Methods

### Plant material and salt-stress treatment

Tunisian barley (*H. vulgare*) landraces with contrasting salt-stress tolerance, Barrage Malleg (tolerant) and Saouef (sensitive) (Ben Chikha *et al.* 2016; Ben Chikha 2017), were used in this study. All experiments were conducted in a glasshouse under controlled conditions (16/8 h day/night photoperiod, temperature of 24 ± 2 °C, light of 270 μmol of photons m^−2^ S^−1^ and a relative humidity of 55–65 %) according to our previous method (Ben Chikha *et al.* 2016). Barley seeds were surface sterilized for 5 min with 5 % sodium hypochlorite and then thoroughly rinsed with distilled water. Ten seeds were sowed in 5 kg polyvinyl chloride (PVC) pots and filled with pre-oven-sterilized (4 h at 200 °C) inert sand (Quarry of Bouarada; Siliana Governorate-Tunisia). All pots were irrigated for 15 days with distilled water (0 mM NaCl) until the emergence of the barley second leaf (10 days old). Pots were then irrigated with Hoagland solution, 100 mL per day per pot (Hoagland and Arnon 1950). One week later when the third leaf was completely expanded, 200 mM NaCl was applied gradually by adding 50 mM NaCl per day to avoid damage of salt-stress shock. Control plants were irrigated with standard growth solution. A completely randomized design with three replications was followed.

### Sampling and growth assessment

All measurements were done on 9 days after 200 mM NaCl treatment. Leaf and root tissues of barley seedlings under salt stress and control conditions were considered. The samples were washed with distilled water to remove soil and other contaminants. For antioxidant enzyme assays and gene expression analyses, three pools of five plants were used. Pooled samples were collected, ground in liquid nitrogen and stored at −80 °C.

Leaf and root length (LL and RL), leaf and root fresh weight (LFW and RFW), and leaf and root dry weight (LDW and RDW) were measured after harvest. Fresh weights were recorded, and then samples were oven dried at 70 °C for 48 h to measure dry weight.

### Proline content

Free proline content was spectrophotometrically determined as described elsewhere (Abraham *et al.* 2010). Oven dried leaf and root samples (100 mg) were homogenized in 3 mL 3 % (w/v) sulfosalicylic acid and centrifuged at 13 000 rpm for 10 min. Then, 1 mL of the supernatant was added to 1 mL of acid-ninhydrin and 1 mL of glacial acetic acid and heated at 100 °C for 1 h. The reaction was cooled in an ice-bath and 2 mL of toluene was added followed by incubation in the dark for 30 min. The upper phase was separated, and absorbance was measured at 520 nm using a spectrophotometer (Spectro UV–Vis Dual Beam PC, UV-S-2007; LABOMED, Inc., Los Angeles, CA, USA). Proline content expressed in µg mg^−1^ DW was determined using a standard curve, ranging from 10 to 100 g mL^−1^, obtained from L-proline.

### Soluble sugar content

Soluble sugar content was measured in leaves and roots using the phenol sulfuric acid method (Dubois *et al.* 1956). Oven dried samples (100 mg) were homogenized with 5 mL hot ethanol (80 %). After filtration, 1 mL of extract was mixed with 0.5 mL of 5 % phenol solution and 2.5 mL of 98 % sulfuric acid. After 1 h at room temperature, the absorbance was measured at 490 nm and the amount of total soluble sugar was calculated using standard curve obtained from glucose as reference and expressed in µg mg^−1^ DW.

### Lipid peroxidation

Lipid peroxidation was expressed as MDA content produced by the thiobarbituric acid (TBA) reaction (Gharibi *et al.* 2016). Frozen leaf and root samples (500 mg) were homogenized in 5 mL of 0.5 % (w/v) thiobarbituric acid solution (TBA) (containing 10 % trichloroacetic acid (TCA)) and heated for 30 min at 95 °C. The reaction was stopped in an ice-bath, centrifuged at 10 000 rpm for 10 min at 4 °C, and the absorbance of the supernatant was measured at 532 nm and corrected for non-specific turbidity at 600 nm. MDA content was determined using the extinction coefficient of 155 mM^−1^ cm^−1^ and expressed as μmol g^−1^ DW.

### Antioxidant enzymes assays

All enzymatic activities were extracted from 500 mg frozen leaves and roots, previously ground in liquid nitrogen, in 50 mM phosphate buffer (pH 7.8) added with 1 % polyvinylpolypyrrolidone (PVPP), 0.1 mM ethylenediaminetetraacetic acid (EDTA), 1 mM phenylmethylsulfonyl fluoride (PMSF), 0.1 % triton X-100 and 10 mM Dithiothreitol (DTT). For ascorbate peroxidase (APX) activity assessment, 5 mM ascorbate was added to the extraction buffer. Extracts were clarified by centrifugation (30 min at 15 000 rpm, 4 °C) and the resulting supernatant was collected. Protein content (μg μl^−1^) was determined (Bradford 1976) and assayed using a UV/VIS spectrophotometer.

Superoxide dismutase (SOD) activity was detected measuring the inhibition of nitro blue tetrazolium chloride (NBT) photoreduction by the enzyme (Del Longo *et al*. 1993). The reaction mixture contained 50 mM phosphate buffer (pH 7.0), 0.1 mM EDTA, 13 mM L-methionine, 50 mM (4-(2-hydroxyethyl)-1-piperazineethanesulfonic acid) HEPES, 50 mM Na_2_CO_3_, 0.025 % triton X-100, 75 μM NBT, 2 μM riboflavin and 20 µL enzyme extract. For NBT photoreduction, reaction tubes were placed under 15 W fluorescent lights for 10 min at 25 °C and the reaction was stopped by switching the light off. Tubes without enzyme developed maximal colour. The absorbance was read at 560 nm and SOD activity was expressed as unit mg^−1^ protein. A non-irradiated reaction mixture, which did not develop colour, served as blank. One unit of SOD activity was the quantity of enzyme necessary to inhibit 50 % of NBT photoreduction in comparison with tubes without enzymes.

Catalase (CAT) activity was assayed by monitoring the hydrogen peroxide (H_2_O_2_) catabolization by measuring the decrease in absorbance at 240 nm for 1 min (Claiborne 1985). The reaction mixture contained 50 mM phosphate buffer (pH 7.0), 10 mM H_2_O_2_ and 50 µL enzyme extract in a final volume of 1 mL. The extinction coefficient 36 mM^−1^ cm^−1^ was used to calculate the enzyme activity (μmol mn^−1^ mg^−1^). One unit of CAT activity was defined as the amount of enzyme that catalyses the degradation of 1 μmol of H_2_O_2_ per minute.

Ascorbate peroxidase (APX) activity was measured following the H_2_O_2_-dependent ascorbate peroxidation at 290 nm for 1 min (Nakano and Asada 1981). The reaction mixture contained 50 mM phosphate buffer (pH 7.0), 0.1 mM EDTA, 0.1 mM H_2_O_2_, 0.25 mM ascorbate and 10 μL enzyme extract in a final volume of 1 mL. The extinction coefficient of 2.8 mM^−1^ cm^−1^ was used to determine the enzyme activity. One unit of APX activity (μmol mn^−1^ mg^−1^) was the quantity of enzyme required for the oxidation of 1 μmol of ascorbate per min.

### RNA-extraction, first-strand cDNA synthesis and real-time qRT-PCR

Total RNA was extracted from 100 mg of frozen leaf and root samples using the ZR Plant RNA MiniPrep™ Kit (Zymo Research, Irvine, CA, USA). RNA quantification and quality were determined by agarose gel electrophoresis and NanoDrop UV5Nano (LabX ready, Kowloon, Hong Kong). RNA samples were cleaned from DNA contamination by RQ1 RNase-free DNase Kit (Promega, Madison, WI, USA) and cDNA was synthesized from 1µg of RNA using GoScript™ Reverse Transcription System Kit (Promega, Madison, WI, USA). All subsequent procedures were performed following the manufacturer’s instructions.

Quantitative Real-Time PCR (qRT-PCR) was performed on 7300 Fast Real-time PCR System (Applied Biosystems, Foster City, CA, USA) using Power SYBR green/ROX qPCR Master mix (Life technologies, Carlsbad, CA, USA). Alpha tubulin (*HvTUB2*) was used as endogenous reference for expression data normalization. Primer sequences of *HvCu/Zn-SOD*, *HvCAT*, *HvAPX1*, and *HvTUB2* genes **[see****Supporting Information—Table S1****]** were designed using Primer 3 software (version 0.4.0) (Rozen and Skaletsky 2000) (http://bioinfo.ut.ee/primer3-0.4.0/). For each gene, the following PCR mix in a total volume of 20 μL was prepared: 1 µL of first-strand cDNA, gene-specific forward primer (200 μM), gene-specific reverse primer (200 μM), 10 μL SYBR Green/ROX and 8 μL H_2_O. The thermal profile was as follows: 95 °C for 10 min followed by 40 amplification cycles of 95 °C for 30 s and 60 °C for 1 min. Melting curves were obtained by slow heating from 65 to 95 °C at 0.5 °C s^−1^ and continuous monitoring of the fluorescence signal. All reactions were performed in triplicate. Quantification of transcript abundance was performed according to 2^−∆∆Ct^ method (Schmittgen and Livak 2008).

### Statistical analysis

All collected data (see Supporting Information—Table S2) were submitted to analysis of variance (ANOVA) to evaluate the effect of salt-stress treatment (T), barley genotypes (G), plant tissue (Pt; leaves and roots) and their respective interactions for all measured traits. For all traits, three replicates were used for ANOVA, correlations and regression analysis. For growth traits the three replicates used were the means of three independently repeated measurements. Stepwise regression analysis was implemented to discover the relationship among traits, and calculated using relative trait changes as ((control − salt stress)/control) for all measured traits. All data are presented as mean ± standard error (SE). Statistical analyses were performed using STATISTICA 12 (StatSoft, Inc., Tulsa, OK, USA).

## Results

### Impact of salt stress on growth and osmoregulation

**Figure 1.**
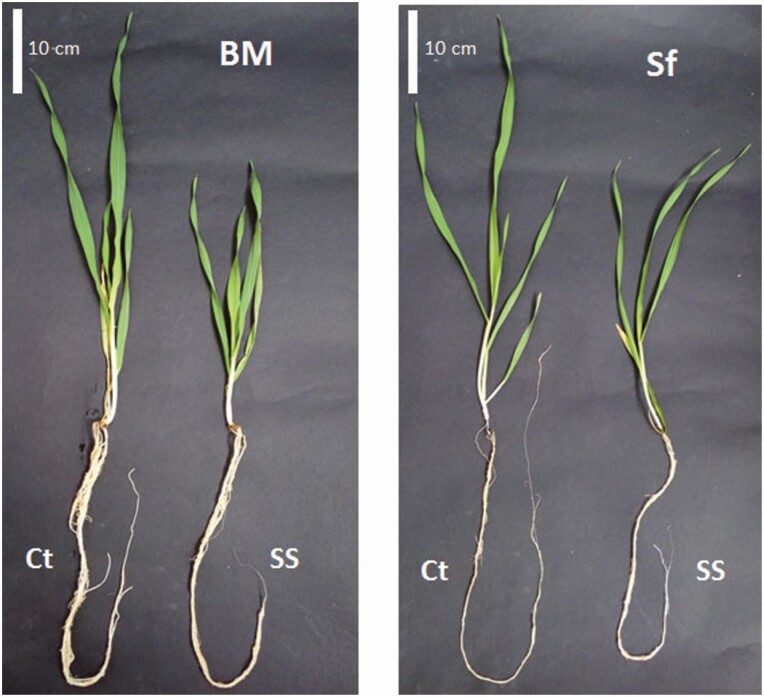
Comparative phenotype of Barrage Malleg (BM) and Saouef (Sf) genotypes under control conditions (Ct) and 200 mM salt stress (SS) for 9 days.

**Figure 2.**
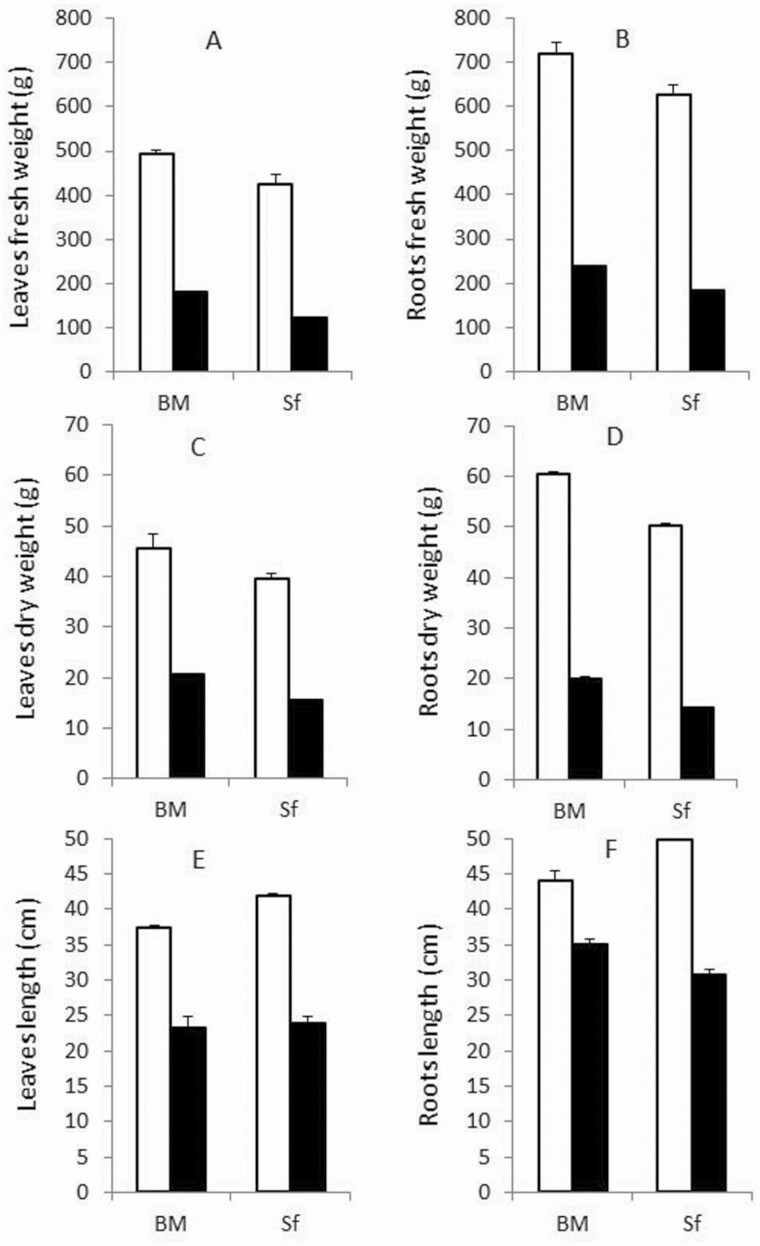
Growth responses and agronomical characteristics of two barley genotypes (Barrage Malleg, BM and Saouef, Sf) treated with 200 mM NaCl for 9 days. (A) Leaves fresh weight; (B) roots fresh weight; (C) leaves dry weight; (D) roots dry weight; (E) leaf length; (F) root length. Open bar: control; closed bar: salt stress.

Salt stress negatively affected all measured growth traits of both barley genotypes (Fig. 1). Growth parameter decreases were more pronounced on the sensitive genotype Sf compared with the tolerant BM, with the exception of FW, where the average decrease was similar in BM and Sf, 64.5 and 70 %, compared with control plants, respectively (Fig. 2A and B).

The decrease in RDW was more pronounced compared with LDW, with a maximum reduction of 71 % for the sensitive genotype Sf compared with control (Fig. 2C and D). Leaf length was affected more than RL with decrease levels in LL by 40 %, for both BM and Sf compared with their respective controls. However, RL was more discriminating between both genotypes, with 20 and 38 % decreases for BM and Sf under 200 mM NaCl compared with control (0 mM NaCl), respectively (Fig. 2E and F).

ANOVA analysis showed that the genotype (G), the salt-stress treatment (T) and the plant tissues (Pt) had significant (*P* < 0.01) effects on all growth traits (Table 1). The interactions of G × T, G × Pt, T × Pt and G × T × Pt were significant (*P* < 0.01) for all those traits except plant length, which was only under the effect of G × T (Table 1).

**Table 1.** Descriptive statistics and analysis of variance explaining effects of genotype (G), treatment (T) and plant tissue (Pt) and genotype by treatment (G × T), genotype by plant tissue (G × Pt), treatment by plant tissue (T × Pt) and genotype by treatment by plant tissue (G × T × Pt) interactions on the major agronomical traits (fresh weight (FW, g), dry weight (DW, g) and plant length (*L*, cm)); proline (µg mg^−1^ DW), soluble sugars (µg mg^−1^ DW), MDA (nmol g^−1^ DW) content, enzymes activities (SOD, CAT, APX) and the expression levels of their respective genes (*HvSOD*, *HvCAT*, *HvAPX*) of two barley genotypes Barrage Malleg (BM) and Saouef (Sf) grown under control and 200 mM salt-stress treatment for 9 days. Significance levels p, **P < 0.01; *P < 0.05; Ns: not significant.

	FW	DW	L	Proline	Soluble sugars	MDA	SOD	CAT	APX	*HvCu/Zn-SOD*	*HvCAT*	*HvAPX1*
Genotypes												
BM	410.28 ± 227.41	36.71 ± 17.96	34.53 ± 7.64	1.03 ± 0.339	332.72 ± 147.40	9.00 ± 0.321	2.03 ± 0.661	0.43 ± 0.288	9.17 ± 6.896	1.76 ± 0.688	1.39 ± 0.517	1.53 ± 0.520
Sf	337.63 ± 206.43	30.01 ± 16.13	37.06 ± 10.96	1.06 ± 0.149	224.56 ± 87.65	10.20 ± 0.416	1.47 ± 0.341	0.38 ± 0.242	8.92 ± 6.345	1.40 ± 0.358	1.46 ± 0.546	1.43 ± 0.446
*Means*	373.95 ± 215.62	33.36 ± 17.04	35.80 ± 9.33	1.05 ± 0.256	278.65 ±130.83	9.60 ± 0.312	1.75 ± 0.588	0.40 ± 0.261	9.05 ± 6.482	1.58 ± 0.568	1.43 ± 0.521	1.48 ± 0.477
Treatments												
Control	566.25 ± 121.29	49.02 ± 8.02	43.50 ± 5.35	0.83 ± 0.135	189.70 ± 75.76	8.80 ± 0.357	1.33 ± 0.263	0.29 ± 0.150	6.50 ± 4.205	1.09 ± 0.092	0.94 ± 0.057	1.02 ± 0.057
Salt stress	181.66 ± 42.69	17.70 ± 2.80	28.10 ± 4.88	1.27 ± 0.122	367.60 ± 113.08	10.40 ± 0.458	2.18 ± 0.508	0.52 ± 0.303	11.59 ± 7.483	2.06 ± 0.391	1.91 ± 0.225	1.93 ± 0.138
*Means*	373.95 ± 215.62	33.36 ± 17.04	35.80 ± 9.33	1.05 ± 0.256	278.65 ± 130.83	9.60 ± 0.214	1.75 ± 0.588	0.40 ± 0.261	9.05 ± 6.482	1.58 ± 0.568	1.43 ± 0.521	1.48 ± 0.477
Plant tissues												
Leaves	305.91 ± 163.62	30.40 ± 13.13	31.67 ± 8.58	1.00 ± 0.224	347.27 ± 123.72	14.70 ± 0.124	1.91 ± 0.684	0.19 ± 0.047	3.47 ± 1.044	1.61 ± 0.689	1.34 ± 0.476	1.53 ± 0.502
Roots	442.00 ± 245.64	36.33 ± 20.39	39.91 ± 8.44	1.09 ± 0.288	210.03 ± 101.03	4.50 ± 0.248	1.59 ± 0.446	0.62 ± 0.201	14.62 ± 4.352	1.54 ± 0.444	1.52 ± 0.569	1.42 ± 0.465
*Means*	373.95 ± 215.62	33.36 ± 17.04	35.80 ± 9.33	1.05 ± 0.256	278.65 ± 130.83	9.60 ± 0.358	1.75 ± 0.588	0.40 ± 0.261	9.05 ± 6.482	1.58 ± 0.568	1.43 ± 0.521	1.48 ± 0.477
ANOVA												
Genotypes (G)	**	**	**	Ns	**	**	**	**	Ns	**	**	**
Treatments (T)	**	**	**	**	**	**	**	**	**	**	**	**
Plant tissues (Pt)	**	**	**	**	**	**	**	**	**	**	**	**
G × T	**	**	**	**	**	**	**	**	*	**	**	**
G × Pt	**	**	Ns	Ns	**	**	**	*	Ns	**	**	**
T × Pt	**	**	Ns	Ns	**	**	Ns	**	**	**	**	Ns
G × T × Pt	**	**	**	**	*	**	*	*	Ns	**	**	**

**Figure 3.**
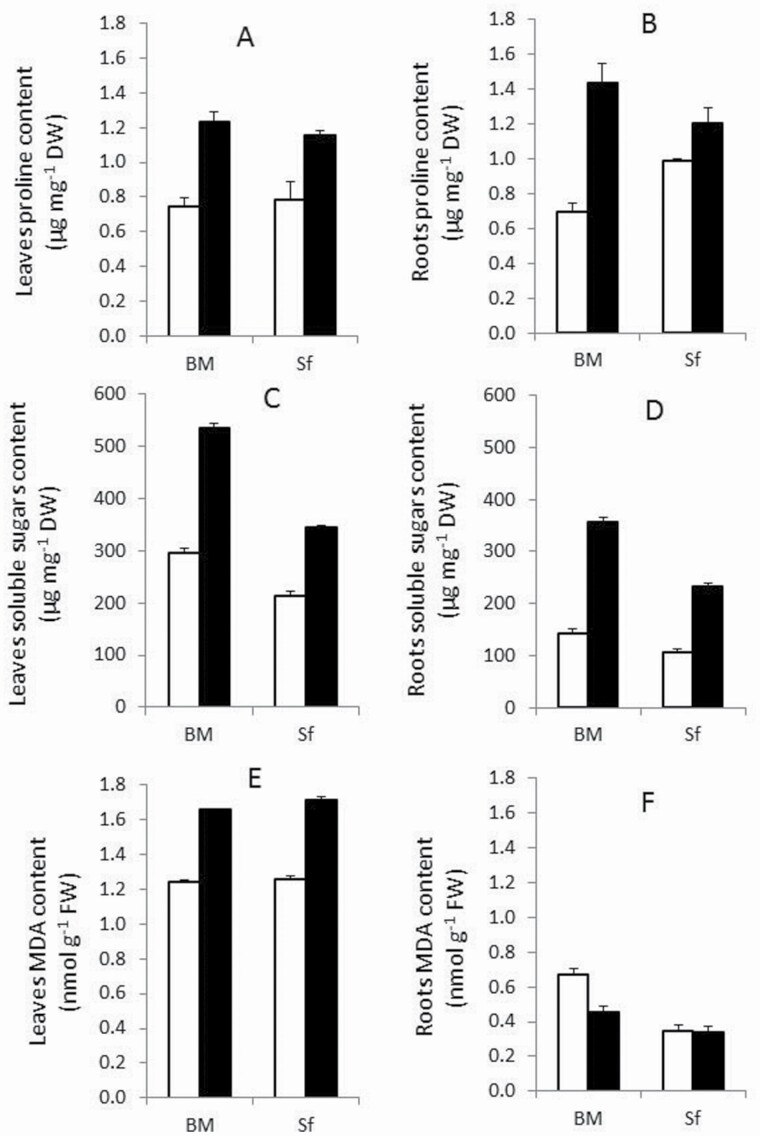
Effects of 200 mM salt treatment on proline (µg mg^−1^ dry weight) (A, B); soluble sugars (µg mg^−1^ dry weight) (C, D); malondialdehyde (nmol g^−1^ dry weight) (E, F); content in leaves and roots of Barrage Malleg (BM) and Saouef (Sf). Open bar: control; closed bar: salt stress.

Salt-stress tolerant BM accumulated more proline and soluble sugars in leaves and roots throughout the entire stress period compared with Sf (Fig. 3). Proline content was significantly (*P* < 0.01) affected by G × T × Pt (Table 1). Salt stress induced a strong increase in proline content in both plant tissues of the salt-stress tolerant genotype BM, with 60 and 81 % in leaves and roots, respectively, compared with control plants (Fig. 3A and B). The salt-stress sensitive genotype (Sf) exhibited a significant increase of 31 and 53 % in proline in leaves and roots, respectively.

Soluble sugars were significantly (*P* < 0.01) affected by interactions with G × T × Pt (Table 1). Strong increases in proline content were detected in BM compared with Sf in both leaves and roots under salt stress (Fig. 3C and D). The average increase of soluble sugars in leaves and roots was 85 % for BM, whereas the increase for Sf was 69 % under salt stress compared with the control.

### Salt-stress effects on MDA content and antioxidant enzymes activities

ANOVA analysis showed that genotype and plant tissue effects were significant (*P* < 0.01) on all antioxidant enzymes activities and MDA content except for APX activity where the genotype effect was not significant. The interactions (G × Pt) and (G × T × Pt) were not statistically significant only for APX activity and the interaction (T × Pt) was not significant only for SOD activity (Table 1).

Salt stress significantly affected leaf MDA content (Fig. 3E) resulting in a similar increase of 35 % in both barley genotypes. Regarding roots MDA content (Fig. 3F) in salt-stress treatment resulted in a significant decrease of 33 % in BM, while the salt-stress sensitive genotype Sf did not show significant variation compared with controls (Fig. 3F).

**Figure 4.**
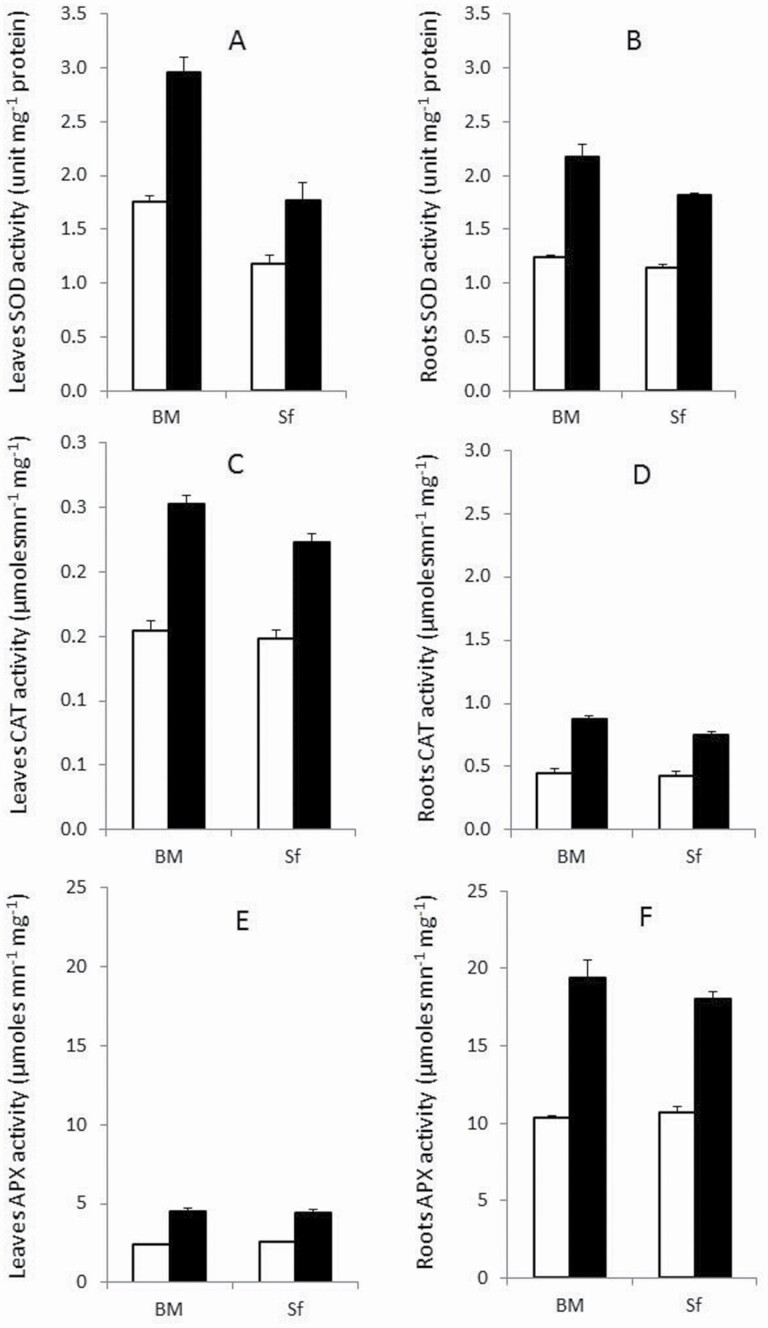
Antioxidant enzymes activities in the leaf (A, C, E) and root (B, D, F) of Barrage Malleg (BM) and Saouef (Sf) genotypes under 200 mM NaCl for 9 days. Superoxide dismutase (A, B); CAT (C, D); APX (E, F). Open bar: control; closed bar: salt stress.

A significant (*P* < 0.01) effect of salt-stress treatment on all antioxidant enzymes activities was detected (Table 1). In fact, salt-stress treatment increased the activities of the measured antioxidant enzymes SOD (66.5 and 60 %), CAT (79.5 and 61.5 %), and APX (88 and 69.5 %) compared with controls in leaves and roots, respectively (Fig. 4).

SOD activity was significantly increased by salt stress in leaf and root tissues of both barley genotypes, with a much higher increase in roots of BM (75 %) and Sf (59 %) compared with control plants (Fig. 4A and B).

The increase of leaf CAT activity was slightly higher in BM tolerant genotype (63 %) compared with Sf (50 %, sensitive genotype). The increase of CAT activity in roots under salt stress was greater in BM than Sf, with 96 and 73 % increase compared with controls, respectively (Fig. 4C and D).

APX activity increase was observed in both genotypes and both plant tissues (leaves and roots). The increase in both tissues was almost identical. The tolerant genotype BM exhibited an APX activity increase of ~ 88 %, while only 70 % was found for the sensitive genotype Sf in both leaves and roots (Fig. 4E and F).

### Effect of salt stress on transcript levels of genes encoding antioxidant enzymes

The expression patterns of *HvCu/Zn-SOD*, *HvCAT* and *HvAPX1* genes were analysed in leaves and roots. ANOVA showed that the interaction effect of G × T × Pt was significant (*P* < 0.01) for all genes (Table 1).

**Figure 5.**
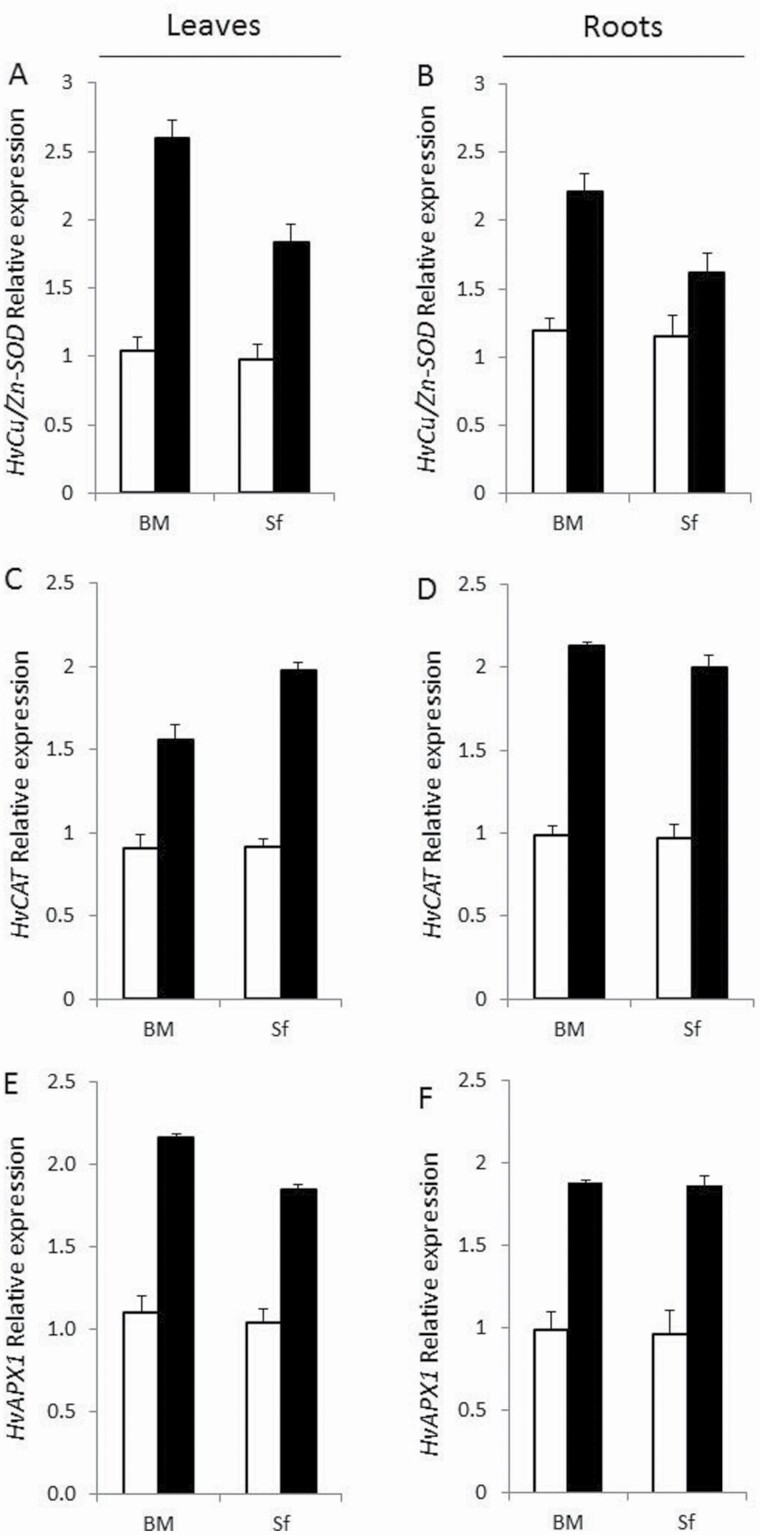
Changes in transcript abundance of genes encoding antioxidant enzymes: *HvCu/Zn-SOD* (A, B); *HvCAT* (C, D); *HvAPX1* (E, F) in leaf and root of Barrage Malleg (BM) and Saouef (Sf) under salt stress for 9 days. Relative expression levels obtained by qRT-PCR for salt stress-regulated genes (closed bars) were normalized relative to control conditions (open bars) and corrected for expression of control gene (*TUB2*). Error bars for qRT-PCR values are standard deviations (*n* > 3).

All genes were expressed under both salt-stress and control conditions (Fig. 5). Compared with control plants, a higher level of expression was obtained for all genes in leaves and roots of salt-stressed plants, with relatively higher expression levels in BM (salt-stress tolerant) compared with Sf (salt-stress sensitive).

The *HvCu/Zn-SOD* gene expression under 200 mM NaCl was almost the same in leaves and roots. However, the tolerant genotype BM showed a higher increase compared with Sf in both tissues. In leaves, BM showed an increase of 149 % compared with 87 % in SF (Fig. 5A). Regarding roots, Cu/Zn-SOD upregulation was more than twice in the tolerant genotype BM (85 %) compared with the sensitive Sf (40 %) (Fig. 5B).

CAT activity was higher in both tissues of BM compared with Sf. Nevertheless, CAT transcript levels showed a higher increase in the leaves of the salt-stress sensitive Sf (116 %) than salt-stress tolerant BM (73 %) compared with control plants (Fig. 5C). In roots, both BM and Sf exhibited a similar trend of increase in CAT transcript levels (~100 %) compared with controls (Fig. 5D).

Higher upregulation of APX was detected in BM leaves compared with Sf (96 and 78 % in BM and Sf compared with control, respectively) (Fig. 5E). Similar upregulation levels in roots (~90 %) were recorded in both BM and Sf compared with control plants (Fig. 5F).

### Correlation and stepwise analysis

Correlation analysis showed a significant positive relationship between DW and FW (*r* = 0.988**; *P* < 0.01), while a negative correlation was detected between FW and expression of *HvCu/Zn-SOD* (*r* = −0.734**; *P* < 0.01), *HvCAT* (*r* = −0.894**; *P* < 0.01) and *HvAPX1* (*r* = −0.914**; *P* < 0.01) **[see****Supporting Information—Table S3****]**. Regarding enzyme activities, DW showed a negative association with SOD activity (*r* = −0.653**; *P* < 0.01) **[see****Supporting Information—Table S3****]**. Comparison of gene expression levels with respective enzymes activities showed that the highest positive correlation was between *HvCu/Zn-SOD* expression and SOD activity (*r* = 0.898**; *P* < 0.01), while no relationship was detected between *HvAPX1* expression and APX activity. MDA was negatively correlated with the activities of both CAT (*r* = −0.802**; *P* < 0.01) and APX (*r* = −0.830**; *P* < 0.01) **[see****Supporting Information—Table S3****]**.

A stepwise regression analysis was conducted to reveal the most important traits (independent variables) contributing to barley dry weight (Table 2). The traits explaining relative dry weight variation were associated with antioxidant response. The relative DW under stressed conditions ((DW_control_ − DW_stress_)/DW_control_) was under the control of *HvCu/Zn-SOD*, which explained 84.4 % of the total variation. *HvCu/Zn-SOD* associated with CAT activity explained 95.7 % of total relative DW variation.

**Table 2.** Stepwise regression for determining most important traits accounting for the relative dry weight ((DW_Control_ − DW_stress_)/DW_Control_) under salt stress. *R*^2^, coefficient of determination. DW: dry weight, FW: fresh weight, CAT: CAT enzyme activity, *HvSOD*: SOD expression level. Significance levels: **P < 0.01.

Treatment	Traits	*R* ^2^	*P*
(DW_Control_ − DW_Salt stress_)/DW_Control_	*HvCu/Zn-SOD*	0.844	**
	*HvCu/Zn-SOD;* CAT	0.957	**

## Discussion

Increasing salt-stress tolerance in cereals to reduce drastic yield losses under harsh environments is the goal of many breeding programs worldwide. Since tolerance to salt is a complex trait attributed to multiple mechanisms, it is imperative to understand the agronomical, physiological and biochemical salt-stress tolerance mechanisms in barley. Severe salt stress (200 mM) previously used to elucidate phenotypic and physiological differences among salt-stress tolerant and sensitive barley genotypes (Ben Chikha *et al.* 2016) was applied to investigate salt-stress response mechanisms within a short period since barley is more sensitive at early vegetative stages (Maas and Poss 1998). Studies of barley salt-stress response reported that the vegetative stage, characterized by high tillering capacity, could be an efficient indicator of vegetative growth, accumulation of reserves and final grain yield under salt stress (Ben Khaled *et al.* 2012; Ben Chikha *et al.* 2016). Hence, early vegetative stages are very important for evaluating the response of barley genotypes to salt stress.

During early vegetative growth, salt stress negatively affects all growth traits including length and fresh and dry biomass of roots and leaves. After 9 days salt-stress treatment, the salt-stress sensitive Sf genotype shows more pronounced growth depression compared with the salt-stress tolerant BM. Similar effects have also been reported previously for barley (Adem *et al.* 2014).

Root development was affected more than leaves, which may be due to the harmful direct contact of roots with the saline solution. This interferes with normal cell division and expansion, leading to lower growth rates. Root weight and length are maintained in the salt-stress tolerant genotype, suggesting its ability to acquire water and nutrients. This feature is reflected by the pattern of shoot growth in this genotype under salt stress. A similar pattern was observed in barley plants exposed to 150 mM salt stress (Witzel *et al.* 2014), which had decreased total root length.

Growth inhibition is more likely a consequence of reduced water uptake due to the osmotic effect of salt stress since under these conditions preserving cellular water content is the main mechanism of cellular growth (Hasanuzzaman *et al.* 2013; Negrao *et al*. 2017). To reach cellular water balance, osmotic adjustment involving the accumulation of proline and soluble sugars is much more efficient in the salt-stress tolerant genotype. This reflects the overall tolerance estimated by whole-plant agronomical (biomass and length) characteristics. Consistent with previous reports (Chen *et al.* 2007; Wu *et al.* 2013), the tolerant cultivar BM tends to synthesize more proline to maintain tissue turgor. The accumulation of proline content in BM was more than twice as high as in Sf in both roots and leaves. Widodo *et al.* (2009) showed an increase in soluble sugar content in response to salt stress in tolerant barley genotype compared with the sensitive genotype. This corroborates our results that the tolerant genotype BM shows higher increase of soluble sugar content in leaves and roots compared with Sf. Proline and soluble sugars are considered important osmoprotectants, involved in osmotic adjustment, sustaining tissue hydration and protecting membranes and proteins from the damage by ROS (Bornare *et al.* 2013; Acosta-Motos *et al.* 2017).

An increase in ROS accumulation in plant cells is generally induced by salt stress due to oxidative stress leading to membrane damage estimated by lipid peroxidation and the formation of malondialdehyde (MDA) (Chaves *et al.* 2009; Abbasi *et al.* 2016). MDA, an indicator of cellular oxidative damage (Das and Roychoudhury 2014), increased with salt stress in leaves, as previously reported (Imrul Mosaddek *et al.* 2012; Hasanuzzaman *et al.* 2018).

To overcome the deleterious effects of salt induced oxidative stress plants have developed a complex antioxidant system to protect cell structures from excessive levels of ROS (Weng *et al.* 2015; You and Chan 2015). Increased activities of ROS scavenging enzymes have been reported in tolerant genotypes (Abbasi *et al.* 2016). SOD is the initial, critical step against oxidative stress because its activity leads to the detoxification of hydrogen peroxide (H_2_O_2_) and superoxide radicals (Abbasi *et al.* 2016). Following this, the hydrogen peroxide dismutation reaction can be catalysed by CAT and APX into H_2_O and O_2_ (Zhang *et al.* 2015; Zhao *et al.* 2020).

Our results showed a significant increase of antioxidative regulation as a response to salt stress in both leaves and roots. These antioxidative regulations were reflected by both transcript abundances and enzyme activities of SOD, CAT and APX, as previously reported for salt-stress tolerance (Abbasi *et al.* 2016; Al Hassan *et al.* 2017). Thus, antioxidative stress response is an essential component of the salt-stress tolerance mechanism in plants. It is notable that the tolerant genotype shows higher transcript abundances and enzyme activities of SOD in leaves and roots, which may remove more ROS, suggesting the involvement of SOD in salt-stress tolerance. However, no major differences were observed in *HvCAT* and *HvAPX1* expression patterns and heir enzyme activities in leaves and roots among both genotypes, indicating that both genotypes similarly regulate these antioxidant enzymes under salt stress. Thus, the measured time point changes in expression patterns of *HvCAT* and *HvAPX1* cannot be used as key criteria for screening salt-stress tolerance barley genotypes in our experiments. It has already been shown that the expression profile of salt-stress tolerance genes change over time (Munns 2005). It should be noted that only one *APX1* isoform was tested on our expression studies even though the presence of different isoforms of *APX* has been demonstrated (Behrouzi *et al.* 2015).

There is a strong positive correlation between the expression profile of the *HvCU/Zn-SOD* gene and its corresponding enzyme activity, which is not the case for the *HvCAT* and *HvAPX1* and their corresponding enzymes. The consistency detected only between the RNA abundance and the enzyme activity of SOD highlights the crucial role of this primary scavenger enzyme in ROS homeostasis regulation during severe salt stress (200 mM) and its involvement in salt-stress tolerance mechanisms, as previously reported (Gupta and Huang 2014). The absence of a correlation between enzyme activities and *CAT* and *APX1* RNA abundances could be due to the complex regulation mechanisms of gene expression and the fact that the expression of oxidative stress response genes fluctuates between days (Adem *et al.* 2014). Thus, according to our experiments, it appears that at 9 days salt stress the expression patterns of *CAT* and *APX1* cannot be considered appropriate criteria to predict the salt-stress tolerance of barley.

The stepwise regression analysis emphasizes the essential role of *HvCu/Zn-SOD* in plant performance under saline conditions. Under salt stress, the relative DW, frequently investigated as salt-stress tolerance indicator of vegetative growth in cereals (Dadshanil *et al.* 2019), is predominantly determined by the *HvCu/Zn-SOD* gene expression level (*R*^2^ = 0.844^**^), as well as by the association between *HvCu/Zn-SOD* and CAT activity (*R*^2^ = 0.957^**^). This implies that under stress conditions, alleviating oxidative stress and providing additional energy for cell homeostasis is the first priority for plants.

**Figure 6.**
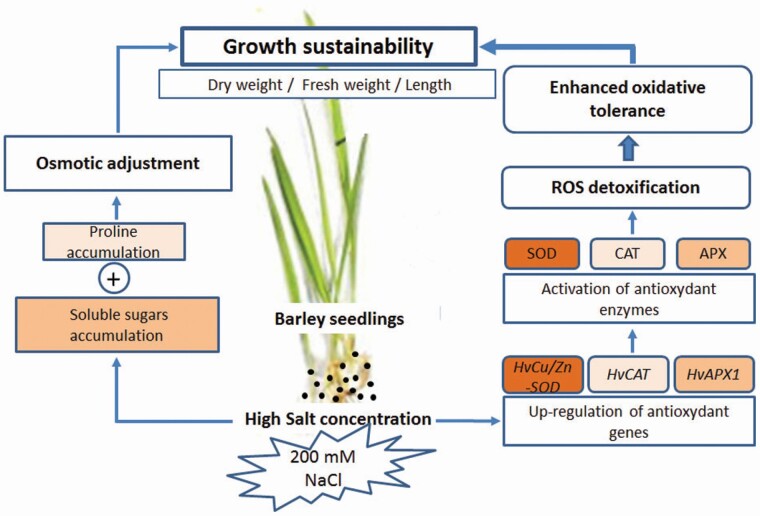
Schematic diagram showing the regulation of growth in barley plants through osmotic adjustment, antioxidant genes expression, and enzymatic activities under salt stress. The colour gradient indicates the level of involvement of the trait from low (light pink) to high (brown).

Overall, salt stress represses plant growth of both analysed genotypes with less reduction in the salt-stress tolerant (BM) genotype than the salt-stress sensitive (Sf) genotype, especially regarding root development. The greater performance of BM over Sf under severe salt stress could be attributed to a better osmotic adjustment and higher ROS scavenging ability, which may play important roles in protecting photosystem machinery and maintenance of growth. Indeed, BM exhibits higher levels of proline and soluble sugars, higher SOD and CAT activities, especially in roots, and more abundance of Cu/Zn-SOD transcripts (Fig. 6).

Osmotic tolerance and ion balance are very important in plant salt-stress tolerance mechanisms. Our findings demonstrate the higher impact of the oxidative tolerance mechanism, especially Cu/Zn-*SOD*, in maintaining barley plant growth under severe saline conditions (200 mM) at the early vegetative stage. The upregulation of Cu/Zn-*SOD* under salt stress and the positive correlation with the activity of its encoded enzyme may protect barley from oxidative damage by reducing the levels of ROS induced by salt stress.

## Conclusion

In summary, 9 days of salt stress imposed by 200 mM NaCl stunted growth of barley plants leading to variable changes of morphological and physiological parameters and inducing osmolyte production, upregulation of expression of genes related to ROS scavenging as well as higher antioxidant enzymes activities. This allowed better osmotic adjustment, alleviating oxidative stress and conferring a deferential performance among tested genotypes. Higher performance was detected on the salt-stress tolerant genotype compared with the sensitive one. Accordingly, tolerance to salt stress at an early vegetative stage was strongly related to osmoregulation as well as detoxification of ROS. This study highlights the protective role of *HvCu/Zn-SOD* against oxidative stress, and indicates expression of this gene is a predominant trait influencing barley early growth under saline conditions. Therefore, a deeper understanding of tolerance mechanisms involving *HvCu/Zn-SOD* expression, activity and related metabolism is critical in the future studies.

## Supporting Information

The following additional information is available in the online version of this article—

Table S1. Primers used for Quantitative Real-Time PCR assays of *HvCu/Zn-SOD, HvAPX1*, *HvCAT* and *HvTUB2* gene expression analyses.

Table S2. Data set.

Table S3. Pearson’s correlation coefficient (*r*) for measured traits from contrasting barley genotypes (Barrage Malleg, BM and Saouef, Sf) under control and salt-stress conditions. SOD, CAT, APX: antioxidant enzymes activities and *HvCu/Zn-SOD*, *HvCAT*, *HvAPX1*; expression levels of SOD, CAT, APX genes; MDA: MDA content; Proline: proline content; SS: soluble sugars; FW: Fresh weight; DW: Dry weight; L: Plant Length. Significance levels: **P < 0.01; *P < 0.05.

plab034_suppl_Supplementary_Table_S1

plab034_suppl_Supplementary_Table_S2

plab034_suppl_Supplementary_Table_S3

## Data Availability

The authors confirm that the data supporting the findings of this study are available as supplementary materials.

## Notes

**Phenome, Genome & Environment. Chief Editor:** Colleen Doherty
