# Supplementary material for: Evaluating the contribution of osmotic and oxidative stress components on barley growth under salt stress
Source: AoB Plants. 2021 Jun 11;13(4):plab034. doi: 10.1093/aobpla/plab034 (PMC8309955; doi:10.1093/aobpla/plab034)
Supplement: plab034_suppl_Supplementary_Table_S1 [file plab034_suppl_supplementary_table_s1.pdf]

Table S1. Primers used for Quantitative Real-Time PCR assays of *HvCu/Zn-SOD*, *HvCAT*, *HvAPX1*, and *HvTUB2* gene expression analyses

Transcript	GenBank accession ID	Forward and reverse primer 5’-3’
HvCu/Zn-SOD	HM537232.1	Forward: 5’- CTTGAAGGACACCGACTTGC -3’<br>Reverse: 5’- CTCAAAAAGCCAAATGACAGTG -3’
HvCAT	U20777.1	Forward: 5’- CTTTCAAGCCAAGTCCGAAG-3’<br>Reverse: 5’- TCCATCCCTGCTGATTAAGG -3’
HvAPX1	AJ006358.1	Forward: 5’- CCAAGGGTTCTGACCACCTA -3’<br>Reverse: 5’- TCAAAGGGTTCCTTGTCCAG -3’
HvTUB2	U40042.1	Forward: 5’- AGTGTCCTGTCCACCCACTC -3’<br>Reverse: 5’- AGCATGAAGTGGATCCTTGG -3’
