# Supplementary material for: Evaluating the contribution of osmotic and oxidative stress components on barley growth under salt stress
Source: AoB Plants. 2021 Jun 11;13(4):plab034. doi: 10.1093/aobpla/plab034 (PMC8309955; doi:10.1093/aobpla/plab034)
Supplement: plab034_suppl_Supplementary_Table_S3 [file plab034_suppl_supplementary_table_s3.pdf]

Table S3. Pearson's correlation coefficient (r) for measured traits from contrasting barley genotypes (Barrage Malleg, BM and Saouef, Sf) under control and salinity conditions. Significance levels p, **: *P*< 0.01; *: *P*<0.05.

	APX	SOD	CAT	*HvAPX1*	*HvCu/Zn-SOD*	*HvCAT*	MDA	Proline	Sugars	FW	DW	L
APX	1	0.051	0.993**	0.280	0.259	0.570**	-0.830**	0.529**	-0.186	-0.126	-0.269	0.076
SOD		1	0.124	0.854**	0.898**	0.557**	0.356	0.646**	0.941**	-0.686**	-0.653**	-0.796**
CAT			1	0.334	0.323	0.610**	-0.802**	0.574**	-0.102	-0.176	-0.313	0.016
HvAPX1				1	0.925**	0.871**	0.261	0.846**	0.815**	-0.908**	-0.914**	-0.889**
HvCu/Zn-SOD					1	0.743**	0.217	0.825**	0.843**	-0.731**	-0.734**	-0.758**
HvCAT						1	-0.054	0.858**	0.501*	-0.837**	-0.894**	-0.710**
MDA							1	-0.011	0.582**	-0.420*	-0.287	-0.532**
Proline								1	0.567**	-0.775**	-0.829**	-0.550**
Sugars									1	-0.737**	-0.668**	-0.850**
FW										1	0.988**	0.874**
DW											1	0.827**
L												1
